# Medical data integration using HL7 standards for patient’s early identification

**DOI:** 10.1371/journal.pone.0262067

**Published:** 2021-12-31

**Authors:** Adi A. AlQudah, Mostafa Al-Emran, Khaled Shaalan

**Affiliations:** Faculty of Engineering & IT, The British University in Dubai, Dubai, United Arab Emirates; Tabriz University of Medical Sciences, ISLAMIC REPUBLIC OF IRAN

## Abstract

Integration between information systems is critical, especially in the healthcare domain, since interoperability requirements are related to patients’ data confidentiality, safety, and satisfaction. The goal of this study is to propose a solution based on the integration between queue management solution (QMS) and the electronic medical records (EMR), using Health Level Seven (HL7) protocols and Extensible Markup Language (XML). The proposed solution facilitates the patient’s self-check-in within a healthcare organization in UAE. The solution aims to help in minimizing the waiting times within the outpatient department through early identification of patients who hold the Emirates national ID cards, i.e., whether an Emirati or expatriates. The integration components, solution design, and the custom-designed XML and HL7 messages were clarified in this paper. In addition, the study includes a simulation experiment through control and intervention weeks with 517 valid appointments. The experiment goal was to evaluate the patient’s total journey and each related clinical stage by comparing the “routine-based identification” with the “patient’s self-check-in” processes in case of booked appointments. As a key finding, the proposed solution is efficient and could reduce the “patient’s journey time” by more than 14 minutes and “time to identify” patients by 10 minutes. There was also a significant drop in the waiting time to triage and the time to finish the triage process. In conclusion, the proposed solution is considered innovative and can provide a positive added value for the patient’s whole journey.

## Introduction

The standards of life and healthcare services for people have been increased due to the development of the economy and the changes in medical systems [1]. People are looking for a higher level of healthcare services with less time to wait. Waiting is not likable by people [2], and in general, waiting time is the most frequent complaint by all patients [3]. Long queues happen in various sectors, i.e., hospitals, banks, and retail stores [2]. In healthcare, taking a long time to book an appointment, get treatment, or take medicine can negatively impact patients’ satisfaction and safety [1, 3–8]. It is essential to have proper solutions for those long queues in healthcare organizations, where these solutions help to manage the queues along with their related statistics. For instance, long waiting times in the emergency department can raise the rates of deaths and admission to hospitals, increase patients’ complaints and reduce productivity. Despite its widely standard design, it is infrequent to capture patients’ arrival time in the triage process [5].

The routine-based process includes a lot of manuals and paperwork, which means more probability of human mistakes. For instance, the receptionist may miss check-in the patient the EMR. In addition, the time to identify the patient is long and not accurately measured, so it can increase the whole journey time, with difficulty or even inability to collect accurate data for statistics and decision-making.

Due to the importance of outpatient departments in hospitals, where all outpatients arrive [9], the purpose of this research is to minimize the long queues and their related waiting times. The study suggests integrating the currently implemented queue management solution (QMS) with the electronic medical records (EMR) solution, using health Level Seven (HL7) protocols. As a pilot study, this research will include a simulation experiment in three of the busiest clinics, internal medicine, orthopedics, and ENT clinics. The new identification process, integration design and components, custom-designed XML and HL7 messages, the conducted simulation, and its related results are thoroughly clarified in this paper. This research seeks to enhance the current process to identify patients’ arrival in the outpatient department in a healthcare organization in the UAE.

## Waiting times in healthcare

Waiting times are significant to assess the patient’s satisfaction and quality of service [10, 11]. Different initiatives regarding the waiting time in healthcare facilities were discussed and applied to solve the issue of long queues, suggest innovative solutions, and facilitate managing the queues with respect to patients’ satisfaction and safety. A survey was conducted by [3] to evaluate the patients’ satisfaction through using electronic kiosks. The study confirmed that waiting time is a common complaint by all patients. Also, it confirmed the importance of waiting time for overall patient satisfaction, along with the staff’s courtesy and the level of cleanliness. On the other hand, a recent study by [5] provided a proof of concept for achieving early identification for patients in the emergency department using a kiosk for self-check-in. Through a trial with control and intervention weeks, the study proved that the proposed solution could significantly reduce the waiting time for patients before getting treatment in the emergency department.

A quick review of the literature shows that various studies were carried out to discuss and propose different methods and solutions for waiting times. However, the main focus of these studies was to evaluate and reduce the waiting times/length of stay only in the emergency departments [5, 12–16]. In contrast, there is a gap in the literature that discusses and solves long waiting times in outpatient departments. Also, the discussed solutions were more related to medical management or business administration. In general, the previously proposed solutions depend on process redesign and management solutions with minimum illustration for the technology and no focus on the technical perspectives. For instance, a quality project to improve the waiting times has took place at the pharmacy in a public hospital in UAE [17]. The project proposed an optimization for using a hospital information system by sending the electronic prescription to the pharmacy once the clinic generates it. The initiative successfully minimized the waiting time from 21.5 to 4 minutes and enhanced the patients’ satisfaction. However, the study did not discuss the technical specifications of the proposed solution.

As well, the work of Alhammadi in [18] has assessed the satisfaction of patients (N = 552) with their waiting time experience in the healthcare facilities in UAE. Also, the study has discussed the strategies to minimize the waiting times in the cases of appointments and walk-ins. The study has encouraged patients for on-time arrival and having more resources to reduce the waiting times. Similarly, the study of [19] has utilized questionnaires to collect responses from 938 healthcare employees in Dubai regarding the root cause for the long waiting times. The study findings focused on the high workload level, availability of facilities, work procedures, and interaction with management as the main causes of long waiting times in healthcare facilities.

Moreover, smart queue management solutions were suggested by the work of [4, 20] to reduce dissatisfaction for patients at hospitals in the UAE. The two articles have discussed the proposed system’s workflow along with the technical aspects of the queue management system on smartphones. Both studies confirmed that the overall satisfaction of patients relies on the spent waiting times.

## Integration and Health Level Seven (HL7)

Nowadays, software applications are mandatory for healthcare professionals to achieve their daily tasks. Information technology reduces the processing time and standardization of protocols to integrate and exchange data. That’s why there is a need to have fast, secure, reliable methods and tools for clinical data transmission within medical informational systems [21]. Various EMR solutions are available in the market (e.g., Epic [22], Cerner [23]. Nevertheless, the same EMR solution is different when it is installed in different healthcare facilities due to its tailored clinical processes, integration with other healthcare solutions [24], and various usage types by healthcare professionals [25]. The identification of common integration processes is possible, but these processes cannot be replicated from one facility to another, so customizations are obligatory [26]. Scholars in [26] have successfully integrated EMR with patient decision aids (PDAs) using HL7 protocols. Also, they confirmed that integration with electronic medical records is complex due to the unforeseen software issues that can be found while troubleshooting, the concerns of data flow security with third party solutions, and the periodic updates of EMR that can cause functionality issues.

The integration between information systems is difficult because it needs to fulfill the business interoperability requirements [27], which is offered by HL7 protocols. HL7 standards are messaging standards where “Level Seven” represents the seventh level which is “application-level” in the seven-layer communications model for Open Systems Interconnection (OSI) in the International Organization for Standardization (ISO). HL7 protocols provide a beneficial standardization for communication interfaces, especially if we consider various applications, different data formats, and the need to transfer and exchange data [21]. HL7 standards are implemented in the healthcare domain and simply work as an application protocol to exchange electronic data. The standards are beneficial for both healthcare providers and IT vendors, and provide the support to exchange, share, integrate, and retrieve the electronic clinical data located in different systems, along with the central patient care solution [28].

HL7 was established in 1987 as a non-profit standards development organization in the healthcare domain. HL7’s key goal is to give everyone the ability to access and utilize accurate health data at the right time and place in a secure manner. HL7 standards arguably are the most widely implemented and used standards with HL7 V2 and V3 standards. HL7 V2.x is more popular as it is implemented in 35 countries worldwide, within 95% of the healthcare organizations in the USA, and it was accredited in 1994 [21] by the American National Standards Institute [29]. The standards are being supported by more than 1600 corporate members in 50 different countries with 500+ members who represent government authorities, pharmaceutical corporations, healthcare providers, and consulting firms [28].

For 30 years, HL7 version 2 has been the major used standard for exchanging healthcare administrative and clinical data. Healthcare information systems use the HL7 v2 protocol to develop standardized interfaces to connect with other systems and exchange data. HL7 covers a broad spectrum of domains, including Patient Administration, Laboratory Orders and Results, and Public Health Reporting. The base HL7 v2 standard [28] is a framework that contains many message events. Each event provides an initial template (starting point) that is intended to be constrained for a specific use case. The application of constraints to a message event is referred to as profiling [30, 31]. Although the HL7 standards were applied and utilized in most healthcare facilities, there is still a notable lack of software solutions that rely on HL7 standards to exchange data among the medical applications [32]. Consequently, this research aims to study the integration between QMS and EMR using HL7 standards to exchange the data of patients and appointments.

## Methods

### Study design and setting

This study contains two main stages. The first stage is responsible for improving the patient identification process and integrating the QMS with the EMR solution (patient’s self-check-in solution). It includes an analysis of the patient journey (current process), proposes the improved business process (To Be), along with its technical perspectives. The second stage of the study is to conduct a simulation experiment to evaluate the feasibility of the proposed patient’s self-check-in solution before it can be used in other clinics. In March 2020, the experiment took place for two weeks (control and intervention) in the internal medicine, orthopedics, and ENT clinics. Those clinics were selected since they are the busiest clinics. In addition, 9 AM till 1 PM are peak hours, so the included appointments in the experiment were booked in these hours and for two physicians from each clinic.

### Patient identification process

This section will clarify the “current” and “to be” states for the process of patients’ identification. Patients and clinical staff are struggling due to the long time it takes to identify the patients and serve them.

#### Patients’ journey—Current process

Currently, all patients with appointments are identified by receptionists using the regular pre-triage process. The pre-triage process occurred when several patients are in a queue waiting in front of the clinic reception desk to register their arrival and wait to be sent to triage rooms. The clinic has a desk-based kiosk and ticket printer. The receptionist has to call each patient manually to verify the documents, check-in the patient, and give a ticket (number). This given number is used to call the patient by the triage nurse using the QMS. The patients will be checked in manually in the EMR and prioritized as per their arrival time regardless of appointment, which can negatively impact waiting and treatment times.

After checking all patient’s documents and details, the receptionist will ask the patient to be seated in the waiting area till the ticket number can be seen on the TV screen, then the patient will be sent to the triage room. The triage nurse will take the vital signs, write the medical notes, types of allergies that a patient has, and add all these details in the EMR. Then the patient has to go back to the reception to get another ticket to be called by the physician. So, the patient has to wait again to see the new ticket’s number on the TV. The number will be called by the physician using the QMS in order to perform the required consultation/treatment. When the physician completes the treatment, the patient’s visit should be closed in QMS and EMR to indicate that the patient’s journey is completed in the clinic.

#### Patients’ journey—“To Be” process

**Fig 1:**
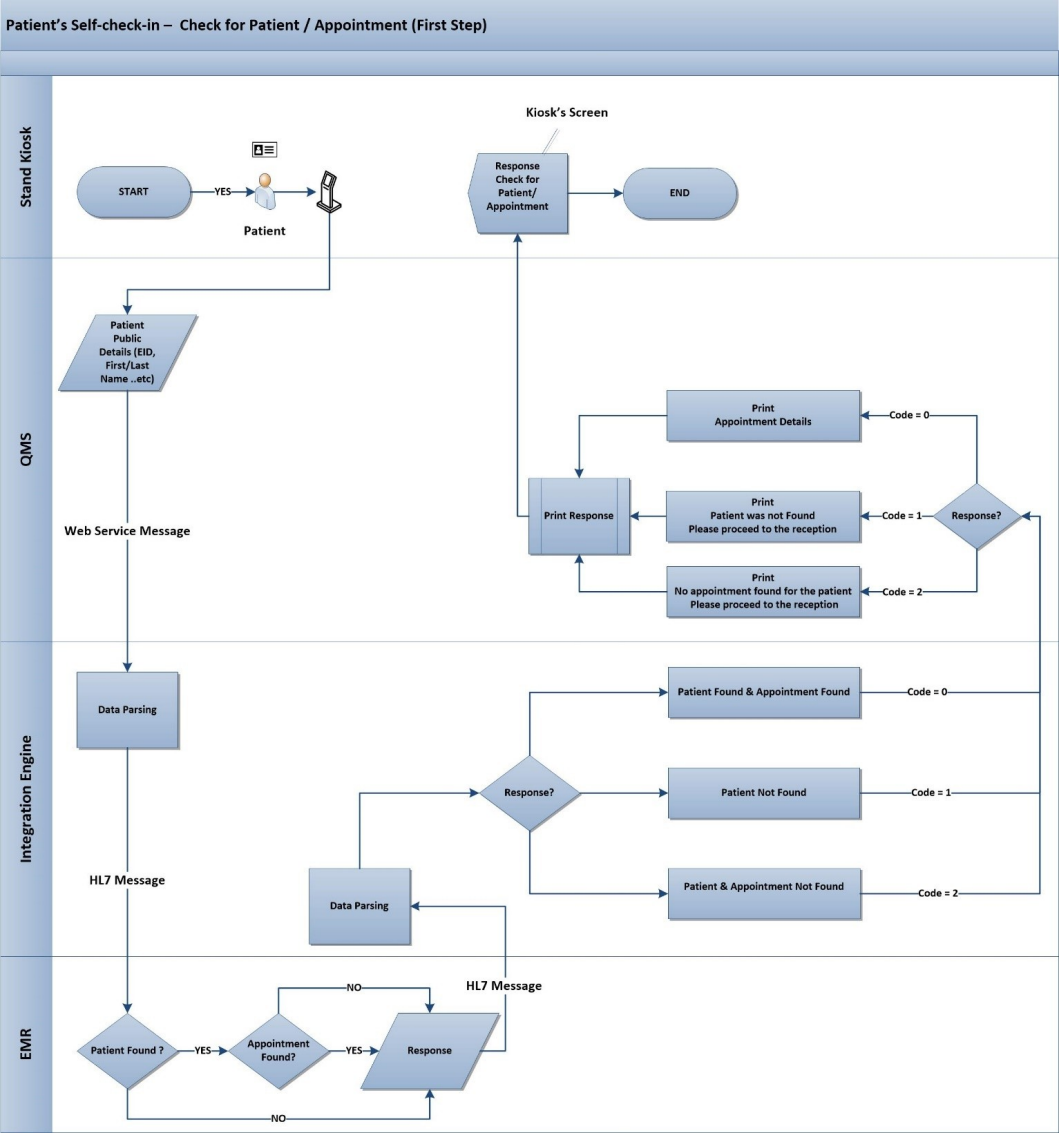
First step—Check for patient/appointment validity.

**Fig 2:**
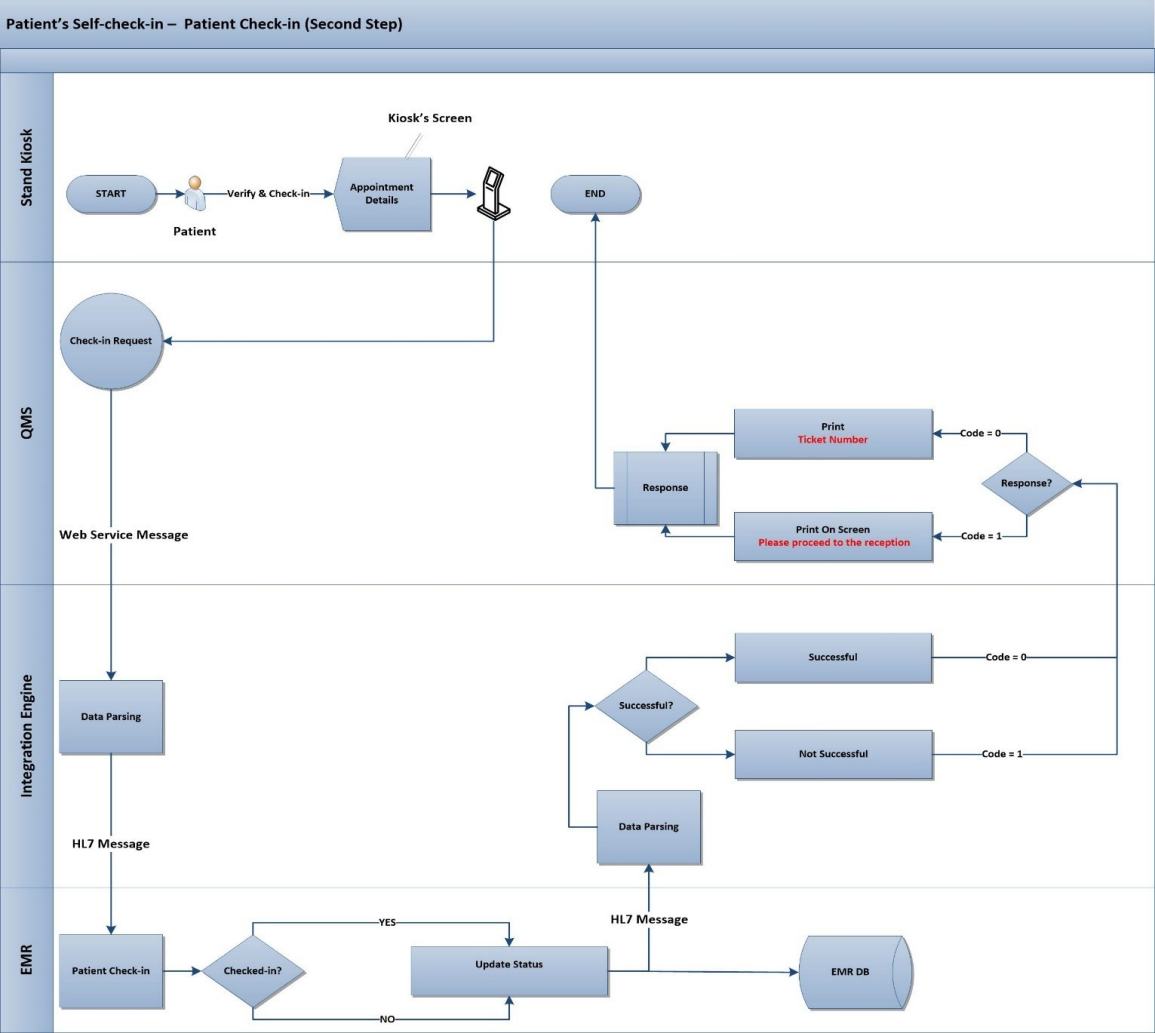
Second step—Patient check-in.

The proposed process to identify the patients with appointments contains two main steps. Those two steps are checking the validity (status) of patient and appointment(s) as in “Fig 1”, and the check-in step using the Emirates ID as in “Fig 2”, in case the first step was succeeded.

The first step will start when a patient arrives at the lobby of the outpatient department (OPD), he/she has to use the stand kiosk that has a “How-to” video to know how to use the Emirates ID and the process to check-in. The patient has to insert the Emirates ID card into the attached smart card reader. Then, the patient’s information will be obtained to check the availability of the patient in the database of the EMR along with his/her appointment(s). The first step will be succeeded if the patient is registered in the EMR, the patient’s details are updated, and has at least one booked appointment. So, a list of booked appointment(s) will be presented on the kiosk’s screen, the patient can click on the desired appointment, the status of the appointment will be updated automatically in the EMR from “Booked” to “Checked-in”, and a ticket number will be printed. Otherwise, an error message will be shown on the screen, as seen in “Fig 1”, so the patient has to visit the clinic’s reception asking for assistance.

In case of successful check-in (Step 2), the patient has to wait until the given ticket number is called by the triage nurse and shown on the TV screen. After completing the routine triage activities, the nurse will transfer the patient to the physician’s account without providing another ticket number as in the current process. Finally, the patient will be called again by the physician to perform the anticipated treatment or consultation.

#### Proposed solution–Technical perspective

The solution of patient’s self-check-in was designed to enhance the current implemented QMS, and integrate it with the EMR solution. The goal of the solution is to achieve interoperability between QMS and EMR to minimize the spent time to identify the patients, triage them, and reduce the time for the patient’s whole journey in the healthcare organization. The solution was implemented using HL7 standards and an integration engine as a middleware solution. The role of the integration engine is to develop integration interfaces and routes as per the specifications. The integration engine works as a translator for the sent and received messages between QMS and EMR.

**Fig 3:**
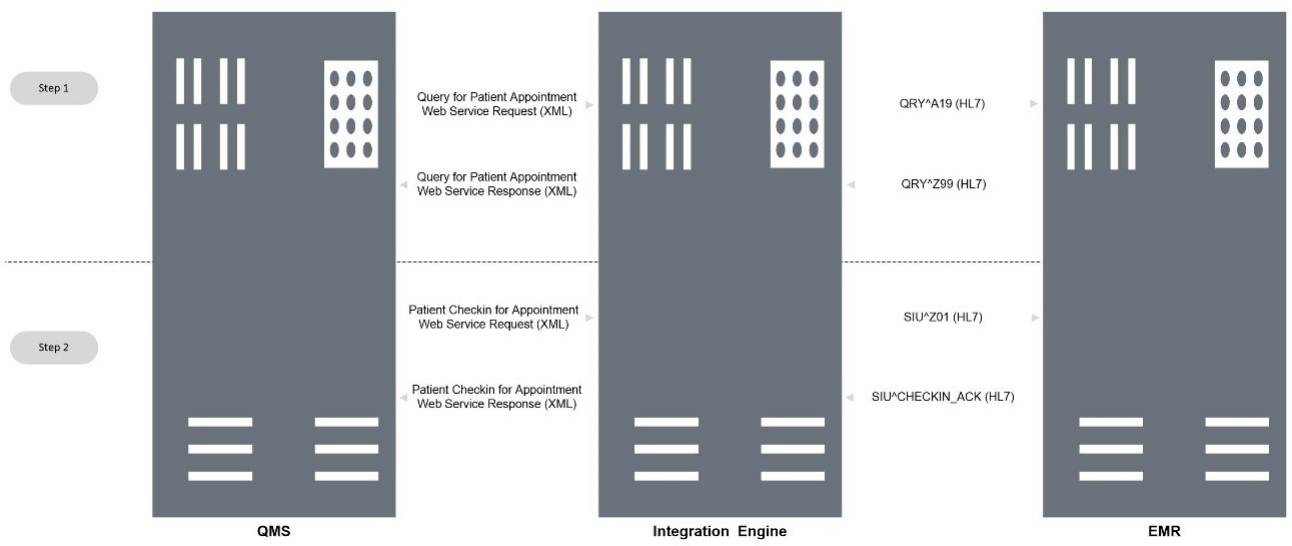
Data flow between integration components.

The designed integration is process-driven, and the integration interface was designed to exchange the data between QMS, EMR, and the integration engine through two different and main steps as seen in “Fig 3”. Each step to have a query-response mechanism with various messages. Where QMS will send and receive XML messages, EMR will send and receive HL7 messages, while the integration engine sends and receives both messages depending on the purpose. To ensure the right process of patient’s self-check-in, the solution was built depending on the patient’s Emirates ID as a unique identifier for each patient.

Once the patient inserts the Emirates ID card into the built-in smart card-reader, QMS will collect the patient’s information, send them through a request (XML message) to the integration engine as seen in “Fig 3”, and the sample message in “Table 1”. Then the integration engine will parse the request, transform it to HL7 message and send to EMR as in second step 2 in “Table 1”. EMR will process the ID number, check if it is available in its database, check the booked appointment(s), and reply back to the integration engine with HL7 message, as the second step in “Table 2”. The reply message will include an acknowledgment code, and the text message depends on the result of the process.

**Table 1:** First step to request patient and appointment details for verification.

**1. Request from QMS to Integration Engine (XML).**
<soap:Envelope xmlns:soap = "http://www.w3.org/2003/05/soap-envelope" xmlns:hco = "HCO"> <soap:Header/> <soap:Body> <hco:getPatientAppointments> <hco:Identifier>784-1234-1234567-1</hco:Identifier> <hco:IdentifierType>National ID</hco:IdentifierType> <hco:FullNameEN>Adi AlQudah</hco:FullNameEN> <!—Optional:—> <hco:Clinic></hco:Clinic> <hco:MaritalStatus></hco:MaritalStatus> <hco:CardNumber></hco:CardNumber> <hco:DOB>01-01-1980</hco:DOB> <hco:Sex>Male</hco:Sex> <hco:Nationality>Jordan</hco:Nationality> <hco:ArabicNationalityAR>الأردن</hco:ArabicNationalityAR> <hco:CardHolderName>عدي القضاة</hco:CardHolderName> </hco:getPatientAppointments> </soap:Body> </soap:Envelope>
**2. Request from Integration Engine to EMR (HL7).**
**Message Template**
**MSH**|^~&|Sending Application| Sending Facility|Receiving Application|Receiving Facility|Date/Time of Message| |QRY^A19|Message Control ID|D|2.3\r **QRD**|QueryDate/Time|Query Format Code|Query Priority|Query ID| | |Quantity Limited Request|ID Number^Family Name^Given Name^^^^^^^^^^Identifier type Code|What Subject Filter\r
• “Query Format Code” was hard-coded to “R”: Response is in record-oriented format. • “Query Priority” was hard-coded to “I”: Immediate. • “Quantity limited request” was hard-coded to “1^RD “: To contain maximum one record • **QRY^A19**: to serve the patient query from another system (EMR). • **QRD.8**: Who Subject Filter, **QRD.8.1**: ID Number, **QRD.8.2**: Family Name, **QRD.8.3**: Given Name, **QRD.8.13**: Identifier type Code.
**Sample Message**
MSH|^~&|ENGINE|HCO|EMR|HCO|20200401122430| |QRY^A19|5431ddb4-d0f9-fera-92a6-8341f83c6d50|D|2.3\r QRD|20200401122430|R|I|Q123456| | |1^RD|784-1234-1234567-1^AlQudah^Adi^^^^^^^^^^National ID|DEM|

**Table 2:** Second step to response with details of patient and appointments.

**1. Response from EMR to Integration Engine (HL7).**
**Message Template**
**MSH**|^~&|Sending Application|Sending Facility|Receiving Application|Receiving Facility|Date/Time of Message| |QRY^Z99\r **MSA**|Acknowledgment Code|Message Control ID|Text Message\r **QRD**|QueryDate/Time|Query Format Code|Query Priority|Query ID| | |Quantity Limited Request|ID Number^Family Name^Given Name^^^^^^^^^^Identifier type Code|What Subject Filter\r **PID**|Set ID|Patient National ID| |Patient MRN|FullNameEn^FullNameAr| |Date of Birth|Sex| | | | | | |Primary Language| | | | | | | | | | | | |Nationality\r **ZAP**|1|Appointment ID|Appointment Type|Clinic Name|Appointment Start Date/Time|Start Time|END Time|Appointment Resource\r
• **MSA.1** - AA: Record found - AE: for any error and the error text to be in MSA.3 • **ZAP**: Repetitive–One per appointment. - **ZAP.2**: Appointment ID. - **ZAP.3**: Appointment Type. - **ZAP.4**: Clinic Name. - **ZAP.5**: Appointment start date time format DD-MM-YYYY HH:MM:SS. - **ZAP.6**: Start time format HH:MM. - **ZAP.7**: End Time format HH:MM. - **ZAP.8**: Appointment Resource (Physician’s name).
**Sample Message**
**MSH**|^~\\&|EMR|HCO|ENGINE|HCO|20200401122431| |QRY^Z99\r **MSA**|AA|5431ddb4-d0f9-fera-92a6-8341f83c6d50|1 Appointment found in the system\r **QRD**|20200401122431|R|I|Q123456| | |1^RD|784-1234-1234567-1^AlQudah^Adi^^^^MR^^^^^^^National ID|DEM \r **PID**|1|784-1234-1234567-1| |121212 |Adi AlQudah^عدي القضاة| |19800101|Male| | | | | | |Arabic| | | | | | | | | | | | |Jordan\r **ZAP**|1|2134732|Internal Medicine FU|HCO Internal Medicine OP|2020-0401-12:30:00|12:30|12:50|Khaled Shaalan
**2. Response from Integration Engine to QMS (XML).**
<soapenv:Envelope xmlns:soapenv = "http://www.w3.org/2003/05/soap-envelope"> <soapenv:Body><hco:getPatientAppointmentsResponse xmlns:hco = "HCO"> <hco:ResponseCode>0</hco:ResponseCode> <hco:AppointmentCount>1</hco:AppointmentCount> <hco:Patient> <hco:MRN>121212</hco:MRN> <hco:EmiratesID>784-1234-1234567-1</hco:EmiratesID> <hco:FirstName>Adi</hco:FirstName> <hco:LastName>AlQudah</hco:LastName> <hco:PatientArabicName>عدي القضاة</hco:PatientArabicName> <hco:Gender>MALE</hco:Gender> <hco:DateOfBirth>19800101</hco:DateOfBirth> <hco:Language>Arabic</hco:Language> </hco:Patient> <hco:Appointment> <hco:AppointmentID>2134732</hco:AppointmentID> <hco:AppointmentType>Internal Medicine FU</hco:AppointmentType> <hco:ClinicName>HCO Internal Medicine OP</hco:ClinicName> <hco:AppointmentStartDateTime>2020-0401-12:30:00</hco:AppointmentStartDateTime> <hco:StartTime>12:30</hco:StartTime> <hco:EndTime>12:50</hco:EndTime> <hco:AppointmentResource>Khaled Shaalan</hco:AppointmentResource> <hco:AppointmentResourceID>Khaled.Shaalan</hco:AppointmentResourceID> </hco:Appointment> </hco:getPatientAppointmentsResponse></soapenv:Body></soapenv:Envelope>

Depending on the received result from EMR (acknowledgment code), the integration engine will parse the message and transform it back to an XML message. Depending on the received result, the new XML message contains a “code” and other patient and appointment(s) details. The new XML file will be sent to QMS, as in the second step in “Table 2” to show a specific message on its screen. If the patient was found and appointment details were retrieved successfully, then the appointment details will be shown on the kiosk’s screen, and the patient has to click on the appointment to start the second step (check-in). Otherwise, the kiosk will show an error message to clarify the case and ask the patient to proceed to the reception counter to solve the encountered issue.

If the first step was successful, the second step would begin when the patient selects the appointment to be checked-in, then QMS will send a new XML request to the integration engine as presented in “Fig 2”, and the first step in “Table 3”. The XML file will be transformed by the integration engine to HL7 with custom trigger-event for appointment check-in “SIU^Z01”, the new HL7 message to be sent to EMR, second step in “Table 3”. Finally, EMR will receive the message, the selected appointment will be checked-in automatically in EMR, and the status will be changed to "checked-in" and updated in the database, EMR will reply with HL7 response that includes the acknowledgment message and successful HL7 message will be sent to the integration engine, as in the first step in “Table 4”, that will pass the code”0” to QMS kiosk to print the ticket as in the second step in “Table 4”. Otherwise, the kiosk will show a message requesting the patient to proceed to the clinic’s reception for additional assistance.

**Table 3:** Third step for “Check-in” request.

**1.Request to Check-in from QMS to Integration Engine (XML).**
<soap:Envelope xmlns:soap = "http://www.w3.org/2003/05/soap-envelope" xmlns:hco = "HCO"> <soap:Header/> <soap:Body> <hco:patientCheckIn> <hco:Patient> <hco:MRN>121212</hco:MRN> <hco:EmiratesID>784-1234-1234567-1</hco:EmiratesID> <hco:LastName>AlQudah</hco:LastName> <!—Optional:—> <hco:PatientArabicName>عدي القضاة</hco:PatientArabicName> <hco:Gender>MALE</hco:Gender> <hco:DateOfBirth>19800101</hco:DateOfBirth> <!—Optional:—> <hco:Language>Arabic</hco:Language> </hco:Patient> <hco:Appointment> <hco:AppointmentID>2134732</hco:AppointmentID> <hco:AppointmentType>Internal Medicine FU</hco:AppointmentType> <hco:ClinicName>HCO Internal Medicine OP</hco:ClinicName> <hco:StartTime>12:30</hco:StartTime> <hco:EndTime>12:50</hco:EndTime> <hco:AppointmentStartDateTime>2020-0401-12:30:00</hco:AppointmentStartDateTime> </hco:Appointment> </hco:patientCheckIn> </soap:Body> </soap:Envelope>
**2. Request to Check-in from Integration Engine to EMR (HL7).**
**Message Template**
**MSH**|^~&|Sending Application|Sending Facility|Receiving Application|Receiving Facility|Date/Time of Message| |SIU^Z01|Message Control ID|D|2.3\r **SCH**|Appointment ID| | | | | | |Appointment Type|||Appointment Timing Quantity:^^^Start Date/Time^ End Date/Time\r **PID**|Set ID|Patient National ID| |Patient MRN|FullNameEn^FullNameAr| |Date of Birth|Sex| | | | | | |Primary Language| | | | | | | | | | | | |Nationality\r
• **SIU^Z01**: Custom trigger for appointment check-in.
**Message Sample**
**MSH**|^~&|ENGINE|HCO|EMR|HCO|20200401122433| |SIU^Z01|08574b23-0bbd-7d80-c1fe-cfad87b5d3a0|D|2.3\r **SCH**|2134732||||||Internal Medicine FU||||^^^20200401123000^20200401125000\r **PID**|1|784-1234-1234567-1| |121212 |Adi AlQudah^عدي القضاة| |19800101|Male| | | | | | |Arabic| | | | | | | | | | | | |Jordan\r

**Table 4:** Final step to acknowledge the success of “Check-in” Request.

**1. Response to Check-in from EMR to Integration Engine (HL7).**
**Message Template**
**MSH**|^~&|Sending Application|Sending Facility|Receiving Application|Receiving Facility|Date/Time of Message| |ACK|Message Control ID|D|2.3\r **MSA**|Acknowledgment Code|Message Control ID\r
• **ACK**: General acknowledgment. • **MSA**.1 - AA: Record found - AE: for any error and the error text to be in MSA.3
**Message Sample**
**MSH**|^~&|EMR|HCO|ENGINE|HCO|20200401122436||ACK|08574b23-0bbd-7d80-c1fe-cfad87b5d3a0|D|2.3\r **MSA**|AA|08574b23-0bbd-7d80-c1fe-cfad87b5d3a0\r
**2. Step 8: Response to Check-in from Integration Engine to QMS (XML).**
<hco:patientCheckInResponse> <hco:ResponseCode> 0 </hco:ResponseCode> </hco:patientCheckInResponse>

### Simulation experiment

#### Control week

The first experiment (control) took place for one week, where the patient will be identified using the usual identification and triage processes (routine). The primary goal of this experiment is to identify and record the time spent to complete each stage within the patient’s journey in the hospital; before applying the new solution. In the control week, the patient’s identification stage is the time interval between the patient’s arrival and the identification by the clinic staff. The patients are identified by receptionists using the regular pre-triage process. Usually, there are three receptionists in the internal medicine and orthopedics clinics, along with three triage nurses, at any time to serve patients. While the ENT clinic has two receptionists and only one triage nurse. Each receptionist has a ticketing solution installed on the computer, while all computers are connected to one thermal ticket printer. The receptionist has to call each patient manually in order to check Emirates ID, documents, and appointment details before giving him/her a ticket (number). Then the process will be followed as illustrated earlier in the current state of QMS. Regularly, patients are given appointments from 8 am to 5 pm, but it was found that there are four peak hours in those selected clinics, from 9 am till 1 pm. So, these busy hours were considered eligible hours to conduct the study, and any appointment booked during these peak hours was included in the study.

#### Intervention week

The second experiment is simply a simulation that took place for one intervention week, where the patient will be requested to bring the Emirates ID and use the self-check-in kiosk. Two kiosks were installed in the lobby of OPD, and each kiosk has a built-in smart card reader. The “To Be” process is to be followed, as discussed earlier. The primary goal of the simulation is to identify and record the spent time to complete each clinical stage within the patient’s journey; after using the new solution. The patient’s identification stage in intervention week can be defined as the time interval between entering the ID to the kiosk’s reader and the identification for the patient by the EMR solution.

Similar to the control week, the simulation took place in the same three selected clinics and considered any appointment that was booked from 9 am till 1 pm.

### Data collection

In the control week, the time of the patient’s arrival to clinic reception was recorded manually by the first researcher with the help of one receptionist in each clinic. The total identification time was calculated after obtaining the ticket printing time from QMS. The time to complete other stages was collected and recorded for each patient through QMS. During the intervention week, the data were extracted from QMS.

### Data analysis

Prior to the statistical analysis, the collected data were studied to classify each stage in each appointment as “within the target” and “out of target”; as per the set of targets recommended by the quality department. Those targets were studied in order to evaluate if the proposed solution will help to improve the performance and achieve targets.

Data statistical analysis was performed using SPSS v.25 [33]. Nonparametric tests were used to overcome any non-normality distribution issue [34]. So, a comparison for “meeting the target” as categorical data was achieved using the classical Chi2 test, and the results were presented as percentages [15]. The two groups (control and intervention) are independent, so the Mann-Whitney U test was employed [34] to compare the differences in median time to complete each stage, along with interquartile ranges [15].

## Results and discussion

After exclusion and analysis for collected appointments’ data, a total of 517 appointments were considered valid to be added to the experiment. Out of 306 total appointments booked in the peak hours for the six physicians in the control week, only 273 (89.22%) were found to be eligible appointments and included in the study. On the other hand, intervention week reported 338 total booked appointments for the six physicians in the peak hours, but only 244 (72.19%) were found to be eligible appointments. There was a total of 94 eliminated appointments, 11 were not included because patients did not bring their Emirates ID, 7 due to staff mistake, while the rest were excluded due to the “No show” state. The high rate of “No show” (22.49%) was driven by the situation of the Covid-19 virus and the precautionary measures and recommendations. All appointments’ characteristics can be seen in “Table 5”.

**Table 5:** Appointment characteristics.

Characteristic	Control	Intervention
**Total Appointments**	306	338
**Average Appointment / Day**	61.2	67.6
**Total Valid Appointments**	273 (89.22%)	244 (72.19%)
**Valid Appointment / Day**	54.6	48.8
**Target–Patient Journey**		
** **Within Target	2 (0.73%)	136 (55.74%)
** **Out of Target	271 (99.27%)	108 (44.26%)
**Gender**		
** **Female	96 (35.16%)	116 (47.54%)
** **Male	177 (64.84%)	128 (52.46%)

The results in “Table 6” show that there is a significant rise in the percentage of “met target” for the identification stage from 0% to 100%. The target was set to be 5 minutes by the quality department, while the mean value for identification in the intervention week was only (μ = 18, σ = 1) in seconds. This indicates a peerless success for the self-check-in solution in the matter of reducing the time to identify the patients. Consequently, the percentage of “met target” for the whole patient’s journey got increased to reach 55.74% in the intervention sample, instead of only 0.73% in the control sample. As well, the improvement of the whole journey in “met target” was achieved because of the significant improvement in “met target” in the case of “Wait to Triage” and “triage” stages. Although it is hard to determine the exact cause of these significant improvements, the authors believe it is likely to refer to the new self-check-in solution. During the intervention week, it was observed that the triage nurses were busy all the time and could call the patients more quickly. There was no need for the triage nurse to sit inactive awaiting the next patient to be checked-in as in the regular process.

**Table 6:** Performance targets–comparison.

Stage	Control		Intervention		Target (min)	Absolute Difference % (95% CI)	**P_value_
	Total*	Met Target % (95% CI)	Total*	Met Target % (95% CI)			
Identification	0	0 (0,0)	244	100 (100, 100)	≤ 5	100 (100, 100)	.000***
Wait to Triage	73	26.74 (21.46, 32.02)	99	40.57 (34.37, 46.77)	≤ 7	13.83 (5.70, 21.96)	.001**
Triage	96	35.17 (29.47, 40.87)	177	72.54 (66.90, 78.18)	≤ 3	37.37 (29.37, 45.37)	.000***
Wait to Treatment	49	17.95 (13.37, 22.53)	38	15.57 (10.99, 20.15)	≤ 10	- 2.38 (-8.84, 4.08)	.471
Treatment	125	45.79 (39.84, 51.74)	118	48.36 (42.05, 54.68)	≤ 15	2.57 (-6.08, 11.22)	.558
Whole Journey	2	0.73 (-0.29, 1.75)	136	55.74 (49.46, 62.01)	≤ 40	55.01 (48.65, 61.36)	.000***

*Total = Number of appointments that met the target.

**Significance level at p***<0.001, p**<0.01, p* <0.05.

On the other hand, the analysis found that the median time to identify patients was 0.3 (0.28–0.32) minutes in the intervention instead of 10.37 (8.90–12.88) minutes for the control patients, so the difference is 10:04 minutes. In addition, it was found that applying the new solution had a significant positive effect on the median time to complete the patients’ journey. The difference in median time for the patients’ journey was found to be 14:11 minutes as in “Table 7”.

**Table 7:** Median time to complete–comparison.

Stage	Control	Intervention	Difference (min)	*P_value_
	Median time to complete (IQR)	Median time to complete (IQR)		
Identification	10.37 (8.90–12.88)	0.3 (0.28–0.32)	- 10:04	.000***
Wait to Triage	8.57 (7.00–11.17)	7.57 (5.55–8.93)	- 01:01	.000***
Triage	3.20 (2.57–3.52)	2.48 (1.93–3.12)	- 00:43	.000***
Wait to Treatment	13.92 (11.53–18.37)	13.60 (11.53–16.79)	- 00:19	.0.259
Treatment	16.07 (14.00–18.87)	15.37 (13.23–17.87)	- 00:42	0.023*
Whole Journey	53.45 (49.65–58.38)	39.26 (36.18–44.13)	- 14:11	.000***

*Significance level at p***<0.001, p**<0.01, p* <0.05.

Despite the drop of 19 seconds in the median time to complete the “wait to treatment” stage, the analysis found that there is also a drop of 2.38 in the percentage of “met target” for “wait to treatment”. Although the results are not significant, it is worth understanding the root cause. The spent time waiting to see the physician is a complex variable that depends on other factors, the specialty of each physician, appointment type (new, follow up, referral…etc.), and the availability of assistant nurse. A significant improvement was observed in the median time to complete the treatment stage (-00:42, P = 0.023), but the treatment stage could not significantly be improved to meet the recommended target (≤ 15 min), and the difference was (2.57, P = 0.558).

Self-check-in is a novel innovative solution to enhance the experience of patients during their journey in the outpatient department within the hospital. The abovementioned findings could prove that solution is feasible to minimize the time to complete the whole journey and other stages. All analysis results are clarified in “Tables 6 and 7”, and the collected data in the control and intervention periods can be found in the supporting information section.

## Implications and future research

This study could provide a number of theoretical and practical implications and insights; to solve the issue of long queues and waiting times in the healthcare domain. Usually, the suggested solutions in other studies are only related to redesign the process or add more staff, with a lack of involvement or explanation for the information technologies. This study could provide a novel innovative solution through integrating multiple technology solutions in healthcare. The contribution of this study can be helpful in highlighting the role of computer science and software engineering in the theory of medical management. The success of such technology solutions can also help the medical staff achieve their tasks efficiently, which may enhance technology acceptance in healthcare and contribute to the theory of technology acceptance. Practically, many other studies have discussed and performed trials related to the long patient’s identification and waiting times in emergency departments or walk-in patients. Instead, the proposed innovative solution in this study helps to provide a practical contribution to solve the issue of a long time to identify the patients with appointments in the outpatient department. This study can be a base for other studies in the future, especially in the field of information technologies integration in healthcare. Also, the study can facilitate other researchers in the field of medical management.

In the future, this study can be extended to include other medical solutions, and other simulation studies can be conducted in other Arab or developing countries using a different design, population, or settings. The study can be expanded by redesigning and automating the patient transfer between triage and treatment rooms. The literature has a gap that needs to be fulfilled by studying the exchange of clinical data and integrating different healthcare information technologies within two or multiple healthcare organizations.

## Study limitations

The study faced different limitations that need to be noted. Firstly, collecting the required business and workflow specifications to change the identification and check-in processes. Secondly, although the sample size was sufficient to perform significant statistical analysis, it was not very large, which may impact the accuracy of the results. The sample size was affected by the limited time (2 weeks) to conduct the experiment. The limited time of the experiment was mandatory to ensure that the investigation will not affect the health services or distract the staff from their tasks. The study took place in March 2020, and the number of appointments was negatively impacted by the precautionary measures of the Covid-19 virus. Finally, the study covers appointments in three clinics only, at one healthcare organization, so the results might not be generalized in other clinics, healthcare organizations, or even other implementations for electronic medical records.

## Conclusion

The main objective of this study was to propose an innovative solution for patients’ self-check-in using Emirates national ID in a healthcare organization in UAE. The proposed solution aims to minimize the spent time to complete the patients’ journey by reducing the time to identify patients and waiting times in the outpatient department.

The study had two main parts. The first one is technical and related to the solution design, data flow through custom-designed XML and HL7 messages. The solution was designed by integrating QMS and EMR solutions using HL7 standards and through an integration engine. The second part of the study included a simulation experiment to evaluate the new check-in process and the feasibility of the proposed solution. A total of 517 appointments were collected, analyzed, and empirically evaluated. The findings of the study indicated that the proposed solution for patients’ self-check-in is appropriate to significantly reduce time to identify patients by the staff and minimize the spent time to complete the patients’ journey. In general, the results provided proof that there is a significant improvement to meet the set targets in the identification, “wait to triage” stages, and the whole journey.

## Supporting information

S1 Appendix Collected data in the control period. (XLSX)

S2 Appendix Collected data in the intervention period. (XLSX)
